# An *ab initio* electronic transport database for inorganic materials

**DOI:** 10.1038/sdata.2017.85

**Published:** 2017-07-04

**Authors:** Francesco Ricci, Wei Chen, Umut Aydemir, G. Jeffrey Snyder, Gian-Marco Rignanese, Anubhav Jain, Geoffroy Hautier

**Affiliations:** 1 Institute of Condensed Matter and Nanosciences (IMCN), Université catholique de Louvain, Chemin des étoiles 8, bte L7.03.01, Louvain-la-Neuve, Belgium; 2 Lawrence Berkeley National Lab, 1 Cyclotron Rd, Berkeley, California, USA; 3 Department of Mechanical, Materials and Aerospace Engineering, Illinois Institute of Technology, Chicago, Illinois 60616, USA; 4 Department of Materials Science and Engineering, Northwestern University, 2220 Campus Drive, Evanston, Illinois 60208, USA

**Keywords:** Electronic properties and materials, Computational methods, Electronic structure

## Abstract

Electronic transport in materials is governed by a series of tensorial properties such as conductivity, Seebeck coefficient, and effective mass. These quantities are paramount to the understanding of materials in many fields from thermoelectrics to electronics and photovoltaics. Transport properties can be calculated from a material’s band structure using the Boltzmann transport theory framework. We present here the largest computational database of electronic transport properties based on a large set of 48,000 materials originating from the Materials Project database. Our results were obtained through the interpolation approach developed in the BoltzTraP software, assuming a constant relaxation time. We present the workflow to generate the data, the data validation procedure, and the database structure. Our aim is to target the large community of scientists developing materials selection strategies and performing studies involving transport properties.

## Background & Summary

Many devices such as solar cells, transistors, and thermoelectric generators rely on materials with specific transport properties such as conductivity, mobility, and Seebeck coefficient. An insight into the electronic transport properties of materials is accessible, although with some limitations, by exploiting the semi-classical approach provided by the Boltzmann theory^1,2^. The transport tensors can indeed be linked to the fundamental electronic structure and atomistic properties of any material using Boltzmann transport theory. From the knowledge of the band structure (e.g., from ab initio computations within density functional theory (DFT)) and of the relaxation times due to different scattering processes, solving the Boltzmann transport equation provides indeed an assessment of the electronic transport tensors^3,4^.

Materials properties can be computed on a unprecedented level using high-throughput (HT) ab initio computing^5–8^. This new paradigm is transforming the way materials are discovered, offering the possibility to select materials with certain properties before going to the lengthy process of synthesis, characterization, and device making^9,10^. Large datasets are also useful to perform data mining studies where trends and correlations between materials properties are revealed^11–14^. Freely accessible high-throughput databases are being built providing data to a large community of scientists to facilitate the screening and data mining process^15^ (the Materials Project^16,17^, Open Quantum database^18^, AFLOW^19^, NOMAD repository^20^, the Harvard Clean Energy Project^21^).

The BoltzTraP code^4^ solves Boltzmann equation by interpolating a band structure computed within DFT and performing all the required integrations. Since its development, BoltzTraP has been used in several high-throughput studies in various fields from thermoelectrics to transparent conducting oxides^22–27^ and also to obtain new general descriptors of the band structure^28^. In this paper, we report on a large dataset of electronic transport properties obtained by combining high-throughput generated ab initio band structures and Boltzmann transport theory within the constant relaxation time approximation. In total, we provide access to the computed electronic transport data for about 48,000 compounds to this date. This is the largest public database of electronic transport data obtained by BoltzTraP and DFT.

This dataset adds to the growing database of materials properties of the Materials Project (MP)^16,17^. It will be accessible via its web interface similar to thermodynamics, battery, elastic^29^, and piezo-electric data^30^. In the remainder of the paper, we summarize the Boltzmann equation within the relaxation time approximation, the properties calculated, and the workflow followed. Finally, we present a graphical overview of the data and compare it with published computational and experimental values to better understand the precision and accuracy of our approach.

## Methods

### Methods definitions

In order to evaluate transport phenomena occurring at the electronic level, a microscopic model of the transport process is needed to assess the transport coefficients of materials. The basic transport equation of the current density in presence of electrical **E** and magnetic **B** field, and a temperature gradient **Δ***T* is $j_{i}=\sigma_{ij}E_{j}+\sigma_{ijk}E_{j}B_{k}+\nu_{ij}\nabla_{j}T+\ldots$. In this work, we limit the development to the first order in the magnetic field **B** and we focus only on the conductivity tensors *σ*_*ij*_, *σ*_*ijk*_, and *ν*_*ij*_.

A semi-classical approach based on solving Boltzmann’s equation, within the relaxation time approximation, is commonly used to describe the conductivity tensors. This model evaluates the electrical conductivity introducing a lifetime, *τ*, for an electron that encapsulates all the different scattering mechanisms that it can undergo^1–3^. Following the notation used in ref. 4 describing the BoltzTraP code, the conductivity tensors can be written as:

(1)σαβ(i,k)=e2τi,kυα(i,k)υβ(i,k)

and using the Levi-Civita tensor^31^ *ε*_*ijk*_:

(2)σαβγ(i,k)=e3τi,k2εγuυυα(i,k)υυ(i,k)Mβu−1,

in terms of the group velocity and the inverse mass tensor:

(3)υα(i,k)=1ℏ∂εi,k∂kα,Mβu−1(i,k)=1ℏ2∂2εi,k∂kβ∂ku.

Apart from the band structure (*ε*_*i*,**k**_), the relaxation time *τ*_*i,***k**_ term needs to be defined. It describes all the scattering processes involved in the electronic transport and, in the most general description, it depends on both energy band index *i* and **k** vector direction. In the section Limitations, we provide a more detailed description about common models used to compute the relaxation time (one of which consist in approximating it by a constant) and how we treat it in our HT approach.

Summing over all the bands and all the k-points in the full Brillouin zone, we calculate a differential conductivity tensor depending on energy: $\sigma_{\alpha \beta }(\varepsilon )=\frac{1}{N}\sum_{i,k}\sigma_{\alpha \beta }(i,k)\delta \left(\varepsilon -\varepsilon_{i,k}\right)$, where *i* is the number of bands and *N* is the number of k-points. The three main transport tensors depending on the temperature *T* and the Fermi level (or chemical potential) of the electrons *μ* are now accessible^4^:

1. the conductivity related to the electric field:

(4)σαβ(T;µ)=1Ω∫σαβ(ε)[−∂fµ(T;ε)∂ε]dε,

2. the conductivity related to the electric and magnetic field:

(5)σαβγ(T;µ)=1Ω∫σαβγ(ε)[−∂fµ(T;ε)∂ε]dε,

3. the conductivity related to the thermal gradient:

$$\nu_{\alpha \beta }(T;µ)=\frac{1}{eT\Omega }\int \sigma_{\alpha \beta }(\varepsilon -µ)\left[-\frac{\partial f_{µ}\left(T;\varepsilon \right)}{\partial \varepsilon }\right]d\varepsilon ,$$

4. the electronic contribution to thermal conductivity:

(6)καβ0(T;µ)=1e2TΩ∫σαβ(ε)(ε−µ)2[−∂fµ(T;ε)∂ε]dε.

where *f*_*μ*_ is the Fermi distribution, Ω is the volume of the unit cell, and *e* the electron charge. From these tensorial quantities, it is straightforward to determine the other following quantities:

(7)κijel=κij0−Tνiα(σ−1)βανβj,theelectronicthermalconductivityatzeroelectriccurrent;

(8)Sij=Ei(∇jT)−1=(σ−1)αiναj,theSeebeckcoefficient;

(9)Rijk=EjindjiapplBkappl=(σ−1)αjσαβk(σ−1)iβ,theHallcoefficient;

(10)n(T;µ)=nυ−1Ω∫g(ε)fµ(T;ε)dε,thedopingcarrierconcentration.

The Seebeck coefficient *S*_*ij*_, also known as thermopower, is one of the characteristic properties of thermoelectrics. Within the constant relaxation time approximation, 1/*R*_*ijk*_ is proportional to the Hall carrier density, a quantity usually obtained in experiments by Hall effect measurements. *n*(*T*; *μ*) is the electron or hole concentration depending on the doping type, calculated via the density of states *g*(*ε*), the number of valence electrons per volume $n_{\upsilon }$ and the Fermi distribution *f*_*μ*_(*T*; *ε*). All these quantities are part of the standard output of the BoltzTraP code.

In addition, we computed the conductivity effective mass. This effective mass is simply derived from the conductivity tensor and the doping carrier concentration:

(11)M¯αβ−1=σαβne2τ

We note that this definition works properly only for semiconductors where the doping carrier concentration (equation (10)) is well defined. In metals and small gap materials it fails because the doping carrier concentration deviates from the total carrier concentration, as we discuss further in the Usage Notes. Effective mass tensors are typically evaluated from band structures by computing second derivatives at a certain k-point (e.g., the valence band maximum or conduction band minimum) along certain symmetry lines through finite differences. There are numerical challenges in doing so^32^ and choosing the k-point to evaluate the effective mass is not obvious when facing band structures with important non-parabolicity, multiple degenerate bands or pockets with close energy in different part of the Brillouin zone. The conductivity effective mass can be also seen as an average over the Brillouin zone and bands of the k-dependent second derivative (equation (3)) as integration by parts leads to:

(12)M¯αβ−1=−∑i∫Mαβ−1(i,k)fµ(εi,k,T)dk4π3∑i∫fµ(εi,k,T)dk4π3.

We note that this conductivity effective mass tensor is dependent on temperature and doping level. This quantity has been successfully used for high-throughput screening of new low effective mass transparent conducting and thermoelectric materials^27,28,33–35^. Hereafter, when we refer to calculated effective mass we mean conductivity effective mass.

The integration of Boltzmann’s equation requires an analytical description of the band structure. The BoltzTraP code provides it using an interpolation method based on a Fourier expansion of the band energies that maintains the space group symmetry by using star functions. The basic idea of this technique is to use more star functions than band-energies, but constraining the number of fit bands $\tilde{\varepsilon }$ to be equal to the number of energy bands *ε* and using the additional freedom to minimize a roughness function *ρ*. This method was introduced by Shankland^36^, verified and tested by Koelling and Wood^37^, and modified by Pickett *et al.*^38^. The BoltzTraP code has been largely tested over the last decade in different applications ranging from superconductors^39^ to thermoelectric^40–44^ materials, and good agreement has been found with experimental values in several cases^45–47^. From a practical point of view, the BoltzTraP code takes as input the electronic energies for different k-points, previously calculated by a DFT code (or other methods), interpolates the bands, and computes the Fermi integrals for different temperatures and Fermi level. Finally, it returns as output all the transport coefficients, along with other data such as the coefficients of the interpolating function.

Finally, we also would like to mention the BoltzWann code^48^: a recent attempt to interpolate bands using Wannier functions^49^. Although it provides a greater accuracy for the interpolated band structures, (e.g., treating the band crossings better), this method has not been as widely tested as BoltzTraP, and it is difficult to exploit within a HT framework since the automated construction of Wannier functions is still in its early stages^50^.

### Computational parameters

The input data needed to run BoltzTraP are the crystal structure and the electronic band structure on a uniform grid. Both of these inputs are computed using the standard high-throughput density functional theory (HT-DFT) recipe from the MP summarized in refs 51,52. The DFT calculations were performed using the Vienna Ab initio Simulation Package (VASP)^53,54^ using the Perdew-Burke-Ernzerhof (PBE)^55^ generalized gradient approximation (GGA) and adopting the projector augmented-wave (PAW)^56,57^ approach. For transition metal oxides with localized *d* orbitals, the GGA+U method was employed setting the MP standard Hubbard corrections^58,59^. Most of the structures contained in the MP database originate from the Inorganic Crystal Structures Database (ICSD)^60,61^. The others come from previous high-throughput projects (e.g., a Li-ion battery screening project^51^) as well as from other databases (e.g., the Open Quantum Materials Database^18^). All structures were fully-relaxed (cell and atomic positions) using a two-step procedure, until the energy difference is lower than 0.0005 eV/atoms. All relaxations were performed with spin polarization on and initializing magnetic ions in a high-spin ferromagnetic. For subsequent calculations spin-polarization was retained only when the relaxation results demonstrated non-zero atomically projected magnetic moments. The band structure calculations were determined for standard primitive cells according to the conventions of Setyawan and Curtarolo^19^. A self-consistent static calculation was first performed in order to converge the charge density using a moderate k-point density to sample the Brillouin zone (90 k-points per Å^−3^ (reciprocal lattice volume) for large gap systems (≥0.5 eV) and of 450 k-points per Å^−3^ for those with small gap (<0.5 eV)). The tetrahedron method has been used for the band structure integration over k space in most of the cases. Whenever this method fails, the Gaussian smearing method has been used^51,52^. Then, two non-self-consistent calculations were performed to evaluate the band structures: the first one along symmetry lines as defined in ref. 19 and the second one on an uniform k-point grid (1,000 k-points per Å^−3^ for large band gap systems, i.e., ≥0.5 eV, estimated from self-consistent runs and 1,500 k-points per Å^−3^ for small band gap systems i.e., <0.5 eV). Spin-orbit coupling was not considered in the current study, but could be implemented as a next step to refine the database.

Doping (i.e., introduction of additional carriers either holes or electrons) has a tremendous effect on electronic transport properties. Doping will set the Fermi level (*μ*) and directly influence the values of the transport properties. A first dataset provides all the transport quantities for both n-type and p-type doping at fixed doping levels ranging from 10^16^ to 10^20^ cm^−3^, increasing the doping by one order of magnitude at each step. A second and finer dataset provides the electronic transport properties at various Fermi level energies (on a uniform bin from −1.5 to 1.5 eV around the Fermi level with an energy increment of 0.005 eV), and temperatures (ranging from 100 to 1,300 K with an increment of 100 K). The transport quantities accessible in the two datasets are listed in Tables 1 and 2. We should note that users interested in values for doping levels not within our fixed dopings from 10^16^ to 10^20^cm^−3^ can use the finer dataset to compute more precise doping (see Usage Notes).

### Limitations

Here, we would like to discuss the main approximation which is made in this work: the constant relaxation time. Looking at the conductivity tensor, the relaxation time *τ*_*i,***k**_ is written in the general form as a tensor depending on both the energy and the direction. All scattering events that can influence electron conduction such as impurity scattering, phonon scattering, etc., are included in this parameter^1,2,62^. Considering this term as a constant thus means that it is modeled to be isotropic and not strongly varying at the energy scale of *k*_*B*_*T*. This is a strong approximation that it is known to be far from experimental values for several materials. Many models have been proposed and tested in order to take into account different scattering processes, both empirical^39,63–66^ and first-principles^67,68^. However such models for going beyond the constant relaxation time are more complex and introduce a dependence on further materials properties such as electron-phonon interaction, deformation potential, elastic constants, and dielectric constants. They are therefore more difficult to use on a high-throughput scale for thousands of materials. We should stress that while more accurate approaches exist, particularly for detailed studies of single materials, the constant relaxation time is extremely useful for a first screening and for getting general trends if the user keeps in mind its limitations^23–25^.

As conductivities (thermal and electronic) depend proportionally on the relaxation time within our constant relaxation time framework, we provide those quantities per unit of relaxation time. The user could then simply multiply these values by a constant relaxation time (typically 10^−14^ to 10^−15^ s) to obtain the final transport properties. The Seebeck coefficient does not depend on the relaxation time within the constant relaxation time approximation. We remind though that in this approximation the sign of Seebeck coefficient is wrong for some metals^69^.

Another issue is related to the k-point grid. Its density is quite important for the precision of transport properties calculated by interpolation. A known problem of the Fourier interpolation is the incorrect determination of band derivatives near band crossings. This problem has been analyzed in ref. 38 demonstrating that if the band crossing is not too close to the Fermi level, the derivative and curvature of the bands are not much affected. A possible solution has been proposed by Uehara *et al.*^70^. Also, as mentioned in ref. 4, this problem is localized only along high-symmetry lines. A dense k-point grid will often solve this issue, and since properties are averaged with respect to k-points and bands their accuracy is not affected significantly. When considering a limited number of materials, a very dense k-grid is commonly used. For example, Madsen suggests 64·10^6^/*V* k-points in the full Brillouin zone^24^. Since we are dealing with thousands of materials, the k-point grid used in this project is coarser. It represents a compromise between computational time and accuracy. However, we stress here that we use a validation method (see Validation section) which tests the quality of the band structure interpolation and assesses if the k-point grid is dense enough to avoid any large failure of the interpolation scheme.

Finally, standard density functionals such as the generalized gradient approximation (GGA) used in this work are known to underestimate band gaps. We have found that, in particular, materials for which we predict band gaps less then about 10 k_B_T, but the true gaps are higher than this value, can be subject to larger errors in the predicted properties^23^.

### Workflow

**Figure 1:**
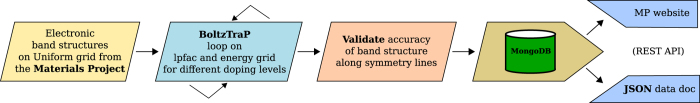
Flowchart illustrating the HT calculation scheme used to calculate and store transport properties.

The sequence of steps used for the HT calculations in order to produce the dataset is illustrated in Fig. 1. It has been automated using the FireWorks workflow software^71^. The Materials Project provides the GGA/GGA+U band structure on a uniform grid for the majority of the materials. On this set of materials, we executed the BoltzTraP code exploiting the BoltztrapRunner class from the pymatgen software^72^. This class, written by some of the authors of this paper, automates writing the four input files required by BoltzTraP, converting units (from eV in Ry and from bohr to Å), checking possible known errors in the output log file, and rerunning BoltzTraP with different parameters in order to solve them. This class also includes an internal loop on two main parameters to get a convergence of the conductivity effective mass. The two tuned parameters in the loop are the *lpfac*, controlling the multiplier for the interpolated mesh and the *energy_grid* that is the increment *dε* used to compute the integral of transport properties. We use another class of pymatgen that we developed, called BoltztrapAnalyzer, to extract the properties from the output and transform them into Python dictionaries that organize the data according to the doping type, doping levels, and temperatures.

Before storing the transport properties, we perform a validation step, which compares the bandstructure on high-symmetry lines calculated by DFT with those interpolated by BoltzTraP. Having a rough assessment of the interpolation accuracy, we can weight the reliability of the related properties. We can also determine in which cases the uniform grid is too sparse, and when needed, recompute the band structure with a denser grid. This validation step is discussed further in the validation section.

Finally, once all the properties are collected for each material, we store them in the form of a JSON (JavaScript Object Notation) data document in the Dryad-repository (Data Citation 1). Furthermore, in the future all currently available data will be accessible via the MP website and obtainable by the MP REST API^73,74^.

### Code availability

The proprietary Vienna Ab Initio Simulation Package (VASP) code^53,54^ is used in this work for the calculation of band structures. The BoltzTraP code is open source and freely accessible. The python classes used to run the BoltzTraP code, extract its output, format it, and perform the accuracy check on bands are implemented in the pymatgen software^72^. Pymatgen is released under the MIT (Massachusetts Institute of Technology) License and is open source. The workflow depicted in Fig. 1 is implemented using the FireWorks software^71^, which is open source under a modified GPL (GNU General Public License). Although VASP is available only under commercial license, the present results can be reproduced by querying for the band structures in the MP database using the associated mp-id and then running BoltzTraP calculations.

## Data Records

The calculated transport properties of ~48,000 materials are reported in the present work. All the considered materials are inorganic solid crystal compounds. Molecules are not included. In order to have an overview of the dataset of structures, we can define two partitions according to the DFT-GGA band gap: about 18,000 metals and about 25,000 semiconductors with band gap higher than 0.1 eV. The calculated transport properties and the associated metadata of all the materials are grouped into two datasets: the first dataset contains higher-level information (the properties listed in Table 1 and the metadata in Table 3); the second dataset contains more detailed information (the properties listed in Table 2). For each material, we provide the transport properties calculated from the GGA band structure (~46,000) and, if available, also from the GGA+U one (~13,000). We stress that both GGA and GGA+U data can be available for the same compound. The two datasets contain a JSON file for each material, grouped in unique compressed archive and stored in the Dryad-repository (Data Citation 1). All the data will additionally be made accessible through the Materials Project website (www.materialsproject.org). The Materials API^73^ and a dedicated web interface of the MP website will be available for downloading the data and querying materials for certain transport properties. The MP website will also include dedicated pages with details for each compound, giving an overview of its calculated properties as well as the calculation parameters.

### File format

The data for each of the calculated material is stored as a JSON document (Data Citation 1). The JSON format is comprised of hierarchical key-value pairs. Tables 1 and 2 report the first level JSON keys, units, the datatype of the values, and a short description, for both datasets. Table 4 contains a description of the dictionary used to store the output of the check of the interpolation of bands. All these keys are inside the main root key called ‘GGA’ (and/or ‘GGA+U’ when available). Table 5 offers a description of the structure of the dictionary used for collecting all the values of each property according to doping type, temperature, doping level, and data type. Additional keys (located at the root level) are provided as metadata for each entry of both datasets. They contain information regarding some of the properties of the materials, such as the crystal structure and a unique mp-id for structure identification within the MP database.

### Properties

The properties included in the two datasets are reported in Tables 1 and 2. Each property is stored in a dictionary and, except for the effective mass, has been calculated for various doping types, temperatures, doping levels, and data type. All these cases are accessible by the sub-keys reported in Table 5.

In the first dataset, the following properties are stored: Seebeck coefficient, electronic conductivity (divided by *τ*), and electronic thermal conductivity (divided by *τ*) for different doping (type and levels) and temperature; carrier and Hall carrier concentration for different temperatures as a function of the Fermi level (energy steps contained as values of the *mu_steps* key); effective masses for a doping concentration of 10^18^ cm^−3^ at 300 K, where n- and p-type refer to electron and hole masses, respectively; the Fermi level values.

In the second dataset (containing additional information intended for expert users) the following properties are stored: Seebeck coefficient, electronic conductivity (divided by *τ*), and electronic thermal conductivity (divided by *τ*) for different temperatures as a function of the Fermi level (energy steps contained as values of the *mu_steps* key); effective masses for different doping levels (n- and p-type) at 300 K; the values of the chemical potential corresponding to each doping level (n- and p-type); the Fermi level values.

In both datasets, for the Seebeck coefficient, the electronic conductivity (divided by *τ*), and the electronic thermal conductivity (divided by *τ*) both the full tensor and its eigenvalues, sorted in ascending order, are stored. For the effective mass, only the sorted (in ascending order) eigenvalues of the full tensor are stored. Regarding the Hall carrier concentration, only the averaged trace of the full Hall tensor is stored. We provide eigenvalues since they are invariant of the axis choice. They are therefore extremely useful to query. For instance, a search for high Seebeck materials would involve a query on the Seebeck eigenvalues. To facilitate queries, the eigenvalues are sorted by ascending order (the first eigenvalue being the smallest one). The anisotropy of a property can directly be assessed by the difference between the last and first eigenvalue. We stress that the provided eigenvalues are sorted in ascending order and do not contain any information about the corresponding principal directions. In order to obtain the correspondence between crystallographic directions and eigenvalues, we suggest to work on the full tensor (and the crystal structure information) and apply an algorithm finding eigenvalues and eigenvectors (see also Usage Notes). We also remind that the effective masses are reported only for semiconductor materials, namely compounds with a band gap higher than zero in GGA or GGA+U.

### Graphical representation of results

**Figure 2:**
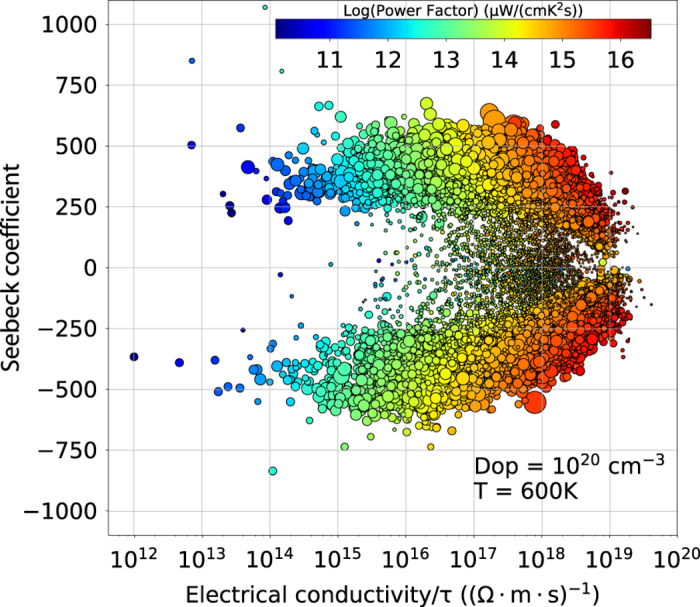
Seebeck versus electron conductivity (divided by *τ*). The color represents the power factor (*S*^2^*σ*) and the pointsize is used for band gap. The reported values are averages over the three direction for T=600 K and n- and p-type doping level (Dop) of 10^20^ cm^−3^. Only materials with band gap higher than 0.1 eV in GGA are considered.

**Figure 3:**
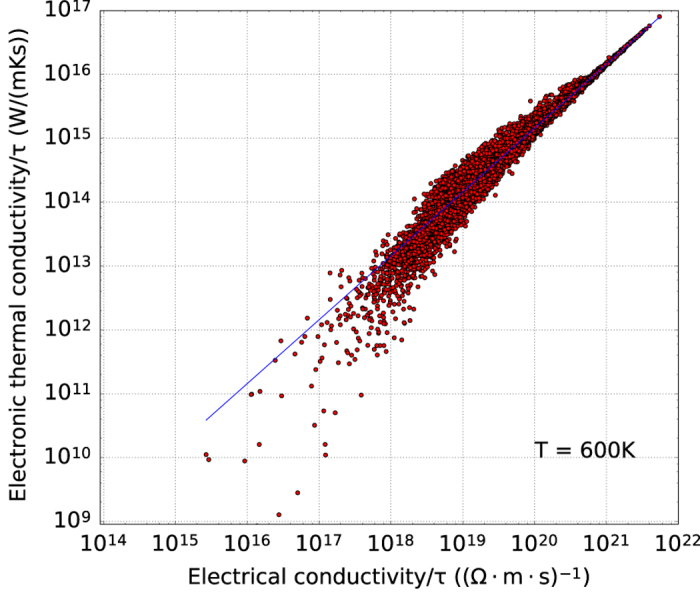
Electron contribution of the thermal conductivity versus electron conductivity: (both divided by *τ*). The values are averages over three direction for T=600 K. Only materials with band gap equal to zero eV in GGA are considered. The blue line represents the Wiedemann-Franz law that holds for metals.

In Figs 2 and 3, we present some of the transport properties stored in the current database. In Fig. 2, we present the Seebeck coefficient as a function of the electrical conductivity (divided by *τ*), for all materials having a GGA band gap higher than 0.1 eV (around 25,000 compounds). Both properties are computed for 600 K and a doping level of 10^20^ cm^−3^. The diameter of the circles is used to indicate the band gap and the color to represent the power factor, *S*^2^*σ* (PF). The graph shows an almost symmetrical spread of points with respect to the x-axis. The two halves contain the two types of doping due to the opposite sign of the Seebeck coefficient. The color gradient shows a reasonable increasing trend toward values of Seebeck and conductivity that maximize the PF. It is evident, however, how difficult it is for materials to reach both high Seebeck and high conductivity at the same time, given the absence of points in that region. The distribution of points according to their size suggests that small band gap materials are concentrated in a range of Seebeck coefficient values lower than 200 *μ*V/K. Above 200 *μ*V/K is difficult to find any trend because of the overlapping of data points.

In Fig. 3, we plot the electrical part of thermal conductivity as a function of the electrical conductivity (both divided by *τ*) for all metallic compounds (with a gap equal to zero in GGA) in the database (~18,000 compounds). For such materials, the electronic contribution of the thermal conductivity can be related to the electrical conductivity and the temperature through the well known Wiedemann-Franz law: *κ*^*el*^/*σ*=*LT*, where *L*=2.4·10^−8^ WΩK^−2^ is the Lorenz number. This law is plotted as a blue line superimposed onto the set of points. The theoretical trend is followed quite well by our dataset, especially for those materials that are common metals with electronic conductivity in the range 10^21^−10^22^ (mΩs)^−1^.

Experimental data for Seebeck, thermal and electrical conductivity stored in the MRL database of thermoelectric properties^75,76^ show very similar trends.

## Technical Validation

### Validation of interpolation precision

Given that the initial uniform k-point grid of band structure might not be sufficient for a good interpolation of all band structures, we performed a post-process check before storing our data. The band structure along symmetry lines given by the interpolation are compared to the one explicitly computed with denser k-point grid which are reported in the Materials Project. This comparison has been implemented in pymatgen.

The comparison is two-fold. First, we assess the correlation distance (as defined in scipy.spatial.distance.correlation class; basically 1−*ρ*, where *ρ* is the Pearson coefficient) between the two energy bands to determine if they behave similarly. Second, we evaluate their energy distance for each segment of high-symmetry path by means of a sum of absolute differences averaged over the number of k-points in each segment: $D_{i}^{k-path}=\frac{1}{N}\sum_{k}\left|\varepsilon_{i,k}^{Bzt}-\varepsilon_{i,k}^{DFT}\right|$, where $\varepsilon_{i,k}^{Bzt},\varepsilon_{i,k}^{DFT}$ are the energies for the band *i* in the k-point *k* calculated by BoltzTraP and DFT, respectively. The output of this check is stored in a dictionary described in the Table 4. It mainly contains the correlation distance and the energy distance (for each segment and for the entire band) for the last (first) four valence (conduction) bands for non-metals or four bands above and four below the Fermi level for metals. For a quick screening, it also contains a warning flag (see ‘acc_err’ key in Table 4), for both correlation distance and energy distance (for the entire band), set to *True* when their average over the eight bands is higher then 0.03. According to this threshold, around 2.5% of GGA/GGA+U band structures have a warning on the correlation and 4% have a warning on the energy distance. The data with a warning on interpolation should be used with extreme caution.

### Validation through comparison to experimental measurements

In this section, we evaluate the level of agreement between calculated properties and the experimental counterpart. Several sources of disagreement can a priori be expected. First of all, we use a series of approximations including DFT, the neglect of temperature effect on the band structure and the constant relaxation time assumption. Numerical effects will also be present in terms of the k-point grid density or the accuracy of derivative close to band crossings although we expect those to be of smaller effect. Finally, experimental measurements are often performed on crystals that could have impurities or be polycrystalline.

Keeping that in mind, we refer to a recent paper by Chen *et al.*^23^ where the Seebeck coefficient and electrical conductivities providing from a same approach using DFT and the constant relaxation time within BoltzTraP are compared with experimental measurements. We summarize here only the main outcomes of the comparison, and refer the reader to the original paper and its supplementary section for more details. The best agreement is by far obtained for the Seebeck coefficient. Mobilities and conductivities are more sensitive to the constant relaxation time approximation but general trends between materials are fairly reproduced. We should stress though that our dataset has not been corrected for the typical band gap error in DFT by a scissor operation.

We finally compare our computed effective mass with experimental data. We only select direct measurements of effective mass through cyclotron resonance and Shubnikov-de Haas (SdH) effect. All the experimental data is obtained from the Landolt-Börnstein database^77^. We take into account the anisotropy of the effective mass when needed and report each symmetrically different direction as a different data point. Our computed effective mass is obtained from the conductivity tensor and averages all the bands contributing to the transport. When compared to cyclotron and SdH measurements of individual bands, we need to average those individual band contributions. We do so by a weighted average following the given formula:

(13)m¯12=m132⋅m1+m232⋅m2m132+m232,

where the individual contributions are labeled with 1 and 2. The formula assumes parabolicity of the bands.

**Figure 4:**
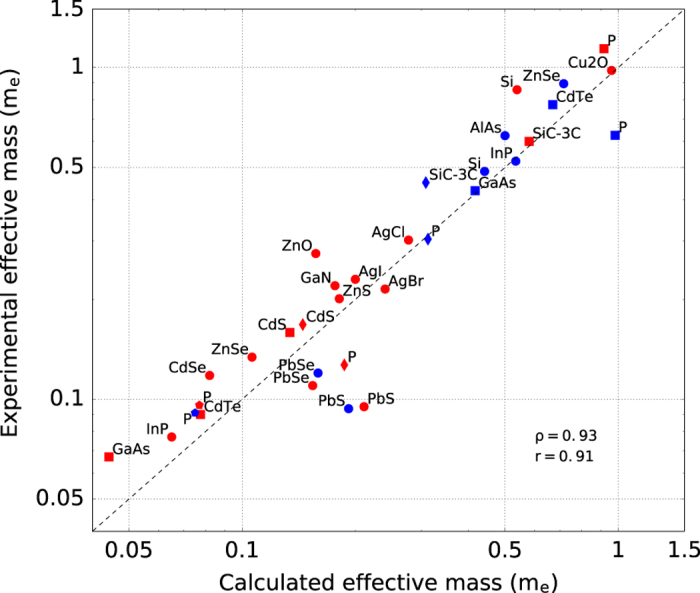
Comparison of effective mass values computed in this work with experimental values from ref. 77. Red and blue colors are used for n and p type materials, respectively. Circles represent the average effective mass values along the three directions. Squares, diamonds, and pentagons are used for effective mass values along x, y, and z directions respectively. Calculated Pearson and Spearman coefficents are equal to 0.93 and 0.91, respectively.

In total we compare 33 effective masses. This is the largest comparison versus experiment to our knowledge. Figure 4 plots the experimental versus the theoretical effective mass obtained by our approach within GGA. The agreement is fairly good and the trends between large and small effective mass materials are well reproduced by DFT. The calculated Pearson and Spearman coefficients are equal to 0.93 and 0.91, respectively. This justifies the use of these DFT effective masses to screen for materials with low effective masses^27,33^. No difference in accuracy between electron and hole effective mass is noticeable. Most of the DFT effective masses underestimate the experimental data. This could come from either a systematic tendency for DFT along the underestimation of the band gap as well for the effect of large polaron present in experiments and not taken into account in our work.

When comparing our results with experiments, one should keep in mind the systematic tendency for semilocal exchange-correlation functionals used within DFT to underestimate the band gap. While the band structure of semiconductors with smaller band gaps can still provides very useful transport properties, the closing of the band gap and the formation of a metallic compounds can lead to much larger deviations.

## Usage Notes

Our paper provides a dataset of transport properties on about 48,000 materials derived from DFT (GGA/GGA+U level) band structures and Boltzmann transport calculations within the constant relaxation time approximation. This type of data has already been used to give insights into fundamental materials properties in electronics, or thermoelectrics. While we warn the user to be always careful in the way this dataset is used (keeping in mind the limits of our approach), this database constitutes a powerful basis for materials search and data mining of materials transport properties.

The meaning of the doping provided by BolzTraP and used in our dataset needs to be clarified. The doping level is not the total amount of carriers. (equation (10)) states that the doping concentration is the difference between the number of electrons per volume present in an undoped material and the number of electrons per volume at the given Fermi level. For a better understanding, we can rephrase it defining the doping concentration as the number of excess holes compared to the number of free electrons at the given Fermi level. It is more clear now that the doping concentration is positive for p-type doping, where there are many more holes than free electrons, and negative for n-type doping, where the opposite is true. We note that mobile carriers that are intrinsically generated, resulting in equal numbers of holes and free electrons, are not considered as part of the doping concentration. For example, metals and small gap materials may include a significant carrier concentration that is intrinsic and separate from the doping levels reported in this work. For such materials, the total carrier concentration can be directly obtained using for instance the Hall carrier concentration. We also remind the user to keep in mind that the Hall carrier concentration does not have to be the same than doping in general. This equality is only exact for parabolic bands when the semiconductor is highly degenerate^78^. When comparing experimental and theoretical results, one should remember that the vast majority of the cases carrier concentration provided experimentally are Hall carrier concentration. Moreover, this definition of carrier concentration affects the assessment of the conductivity effective mass given by equation (11). Therefore we report the effective mass only for materials with an energy gap higher then zero in GGA or GGA+U and we advice the user to be careful using the effective mass for materials with an energy gap lower than 0.1 eV.

As mentioned, we provide in the first dataset all the transport properties at fixed doping levels. If the value of a certain property at a different doping level is needed, it is possible for the user to use the second dataset providing properties in function of Fermi level. When a target doping is set, the user can find what Fermi level would provide this doping level at the required temperature and use the properties corresponding to this Fermi level and given temperature.

In both datasets, we stored both the full tensor and its sorted (in ascending order) eigenvalues for the Seebeck coefficient, the electronic conductivity (divided by *τ*), and the electronic thermal conductivity (divided by *τ*). The eigenvalues (also sorted in ascending order) of the effective mass are also provided. In case the value of a property along a specific direction of the crystal is needed, the use of the full tensor and the structure are mandatory. It is also important to note that when a derived property is needed (e.g., the power factor *S*^2^*σ*), it would be wrong to operate on eigenvalues (since they might not refer to corresponding directions). Therefore, we strongly suggest to instead perform the operations on the full tensors. Eigenvalues can be obtained by running an adequate algorithm on the resulting full tensor.

## Additional Information

**How to cite this article:** Ricci, F. *et al.* An *ab initio* electronic transport database for inorganic materials. *Sci. Data* 4:170085 doi: 10.1038/sdata.2017.85 (2017).

**Publisher’s note:** Springer Nature remains neutral with regard to jurisdictional claims in published maps and institutional affiliations.

## Supplementary Material



## Figures and Tables

**Table 1:** Transport properties stored in the first dataset with their units and data size.

**Transport Property Keys**	**Units**	**Datatype**	**Size**	**Description**
cond_doping	(Ω*ms*)^−1^	array, array	3×3, 1×3	Full tensor and its sorted eigenvalues of the electronic conductivity (divided by *τ*) for different doping (type and level) and temperature
kappa_doping	*W*/(*mKs*)	array, array	3×3, 1×3	Full tensor and its sorted eigenvalues of the electronic thermal conductivity (divided by *τ*) for different doping (type and level) and temperature
seebeck_doping	*μV*/*K*	array, array	3×3, 1×3	Full tensor and its sorted eigenvalues of the Seebeck coefficient for different doping (type and level) and temperature
carrier_conc	*cm*^−3^	float	*n*×1	Doping carrier concentration for different temperature in the range of energy values specified in the *mu_steps* key
hall_carrier_conc	*cm*^−3^	float	*n*×1	The averaged trace of the full tensor of the Hall carrier concentration for different temperature in the range of energy values specified in the *mu_steps* key
cond_eff_mass	*m*_*e*_	array	1×3	Sorted eigenvalues of the conductivity effective mass tensor, at the 10^18^ cm^−3^doping level (n- and p-type) and at 300 K.
mu_steps	eV	array	*n*×1	The Fermi level values.
See Table 5 for the available keys inside each of these root keys. The electronic conductivity and the electronic contribution of the thermal conductivity are stored divided by *τ*. For the electronic conductivity, the electronic contribution of the thermal conductivity, and the Seebeck coefficient both the full tensor and its sorted (in ascending order) eigenvalues are provided.				

**Table 2:** Transport properties stored in the second dataset with their units and data size.

**Transport Property Keys**	**Units**	**Datatype**	**Size**	**Description**
cond_mu	(Ω*ms*)^−1^	array, array	3×3, 1×3	Full tensor and its sorted eigenvalues of the electronic conductivity (divided by *τ*) for different temperature in the range of energy values specified in *mu_steps* key.
kappa_mu	*W*/(*mKs*)	array, array	3×3, 1×3	Full tensor and its sorted eigenvalues of the electronic thermal conductivity (divided by *τ*) for different temperature in the range of energy values specified in *mu_steps* key
seebeck_mu	*μV*/*K*	array, array	3×3, 1×3	Full tensor and its sorted eigenvalues of the Seebeck coefficient for different temperature in the range of energy values specified in *mu_steps* key
cond_eff_mass	*m*_*e*_	array	1×3	Sorted eigenvalues of the conductivity effective mass tensor for n- and p-type and different doping levels at 300 K.
mu_steps	eV	array	*n*×1	The Fermi level values.
mu_doping	eV	float	1×1	The values of the Fermi level for each doping level of the two types of doping.
The electronic conductivity and the electronic contribution of the thermal conductivity are stored divided by *τ*. For the electronic conductivity, the electronic contribution of the thermal conductivity, and the Seebeck coefficient both the full tensor and its sorted (in ascending order) eigenvalues are provided.				

**Table 3:** Additional keys for each entry of both datasets and their descriptions.

**Key**	**Datatype**	**Description**
mp_id	string	IDs for entries in the Materials Project
pretty_formula	string	Chemical formula
cif_structure	string	Relaxed crystal structure represented in Crystallographic Information File (cif)
spacegroup	dictionary	Space group details contained in the followings keys: ‘symbol’, ‘number’, ‘point_group’,’source’,’crystal_system’,’hall’
volume	number	Volume of the relaxed structure in Å^−3^
nsites	number	Number of atomic sites for the conventional cell
gap	dictionary	GGA (and GGA+U when available) band gap. ‘GGA’ and ‘GGAU’ are the keys

**Table 4:** Description of the dictionary structure used to store the output of the interpolation check of bands.

**Root Key**	**1st Level Keys**	**2st Level Keys**	**Keys description**
bands_check			Key of the dictionary containing all the information about the interpolation check of bands
	nb_list		The list of the bands where the check is performed
	avg_corr		The average over all the bands for the correlation
	avg_distance		The average over all the bands for the distance
	‘45’,...		The index of the band
		Corr	Correlation value for the band
		Distance	Distance value for the entire band
		‘Γ−*X*’,...	Distance value only for the high symmetry path segments
	acc_err		List of two Boolean values: *True* if the average of correlation/distance quantity over the eight bands is higher then 0.03; *False* otherwise.
This dictionary is stored in the first dataset. ‘45’ and ‘Γ−*X*’ are examples for the band index and the high symmetry segments keys, respectively. A *True* value in the ‘acc_err’ list has to be considered as a warning of low accuracy in the interpolation of the bands.			

**Table 5:** Description of the dictionary structure used to store transport properties in both datasets.

**Level**	**Keys**	**Type**	**Keys description**
1	n, p	string	Doping type keys available for *‘x’_doping*, *cond_eff_mass*, *mu_doping* root keys.
2	300, 400,..., 1,300	integer	Temperature keys available for *‘x’_doping, ‘x’_mu, carrier_conc, hall_carrier_conc, mu_doping* root keys.
3	1e+16, 1e+17,..., 1e+20	string	Doping level keys available for *‘x’_doping, cond_eff_mass, mu_doping* root keys.
4	tensor, eigs	string	Data type key for *‘x’_doping, ‘x’_mu* root keys.
